# Knowledge, Attitude, and Performance of Nurses toward Hand Hygiene in Hospitals

**DOI:** 10.5539/gjhs.v8n8p57

**Published:** 2015-12-17

**Authors:** Alireza Sharif, Azizollah Arbabisarjou, Abbas Balouchi, Sudabeh Ahmadidarrehsima, Hamed Haddad Kashani

**Affiliations:** 1 Department of Infectious Diseases, Kashan University of Medical Sciences, Kashan, Iran; 2 Health Promotion Research Center, Zahedan University of Medical Sciences, Zahedan, Iran; 3 Department of Medical surgical, Student Research Committee, School of Nursing and Midwifery, Zabol University of Medical Sciences, Zabol, Iran; 4 Anatomical Sciences Research Center, Kashan University of Medical Sciences, Kashan, Iran

**Keywords:** knowledge, attitude, performance, hand hygiene, nurses

## Abstract

**Introduction:**

The proper hand hygiene is one of the foremost techniques to reduce Nosocomial infections. The hand hygiene is deemed as the simplest method for control of Nosocomial infections if it is done properly it may prevent from a lot of costs and fatalities. Due to constant relationship with patients, nurses play paramount role in proper execution of hand hygiene among clinical personnel. The current study was carried out in order to analyze knowledge, attitude, and performance of nurses regarding hand hygiene.

**Materials and Methodology:**

A cross-sectional study was conducted on 200 (of 240) nurses from three hospitals in Kerman city at east of Iran in 2015. The standardized questionnaire was the tool for data collection. These data entered in SPSS (V.22). The frequency and percentage of frequency in descriptive statistics was employed for data analysis. The confidence interval was considered as 95%.

**Results:**

The results showed that the majority of participants were male173 (86.5%), had BA degree 161 (80.5%) and were married 155 (70.5%). Most of nurses 77 (38.5%) had working experience (5-10years). The majority of nurses had good knowledge 149 (74.5%), positive attitude 141 (70.5%) and good performance 175 (87.5%).

**Discussion and Conclusion:**

The nurses are good level in terms of knowledge, attitude, and performance but improvement of their knowledge and knowledge seems to be more necessary by holding educational classes and courses in cases where they have less knowledge.

## 1. Introduction

One of the foremost problems in public health is hospital-related infections in the world (Sarani et al., 2015; Shinde & Mohite, 2014). According to statistics from World Health Organization (WHO), today 1,400,000 patients directly and indirectly suffer from side-effects of Nosocomial infections (Hosseinialhashemi et al., 2015) Prevalence of Nosocomial infections is 40% in developed countries (Asadollahi et al., 2015). while this rate has been reported 9.4% in Iran (Askarian, Yadollahi, & Assadian, 2012). The Nosocomial infections are assumed as the most prevalent reasons for mortality and rising disability among patients (Morrison & Yardley, 2009), which cause increase in period of staying patient in hospital and imposing heavy costs on patients and national health- medical system and eventually leads to mortality of patients (Wilcox & Dave, 2000). The Nosocomial infections mainly take place in special care unit (ICU, CCU, and NICU), acute surgery, and orthopedics (Moody et al., 2013). The greater frequency of such infections in special care units may be due to susceptibility of patients to Nosocomial infections in these wards (Shinde & Mohite, 2014). The majority of Nosocomial infections are transferred through hands of healthcare personnel (Elaziz & Bakr, 2015). One of the paramount techniques to reduce Nosocomial infections is proper hand hygiene (Larson et al., 2000). According to the guideline of WHO, hand is always identified as the first method for prophylaxis but fewer of them advocate it upon execution (Mertz et al., 2011; Park et al., 2014; Polin et al., 2012).

During a study that was done in China, it was shown that paying due attention to improving technique of hand-washing in healthcare personnel during care-giving to risky patients in epidemic spread of acute respiratory syndrome (SARS) might noticeably reduce transferring the infection between personnel with patients (Bennett et al., 2015).

However, we are living in period of knowledge explosion that change of knowledge culture and performance is very fast (Arbabisarjou, 2012). The health authority can improve these changes via life-long learning as in hand hygiene. Whereas hands of nurses are in close contact with patient and they may contaminate patients through daily care-giving including touching, hearing, and by means of instruments (Kampf & Löffler, 2010) thus they play key role in Nosocomial Infections (Elaziz & Bakr, 2015; Joukar & Taherri, 2007). However, one of the ethical and professional important tasks of nurses is to protect from patients with observance of healthcare principles. Based on nursing standards of US nursing association, nurses shall provide the highest level of standard care-giving for patient all the times. Despite of the above cases, nurses are not too inclined observance of healthcare principles and assume it unimportant (Cambell, 2010). The study of Nazarko et al indicated that most of nurses lacked adequate knowledge about hand hygiene and believed they were busy and they could not take their valuable time for washing hand (Nazarko, 2009). Many nurses prefer to wear gloves instead of washing hands and to dispose it after using glove without washing hands and/or they use the same glove for different patients. The other reason for non-observance of hand hygiene is the dermal lesions caused by using detergent and antiseptics (Wilcox et al., 2003). It was shown in another study in Sri Lanka that only 60% of physicians observe principles for washing hands properly (Gunasekara et al., 2009). This may be due to several reasons including shortage of facilities, great number of patients, cultural issues, perception, belief, knowledge, poor performance, short time, and or lesser ratio of number of nurse-to-patient (Pittet, 2001) and other factors may lead to lower performance of nurses including: oblivion (Patarakul et al., 2005), crowd (Barrett & Randle, 2008), and attitude of nurses about this issue that an important factor is available for washing hand (Jenner et al., 2006) the widespread execution of hand hygiene in these wards causes reducing Nosocomial infections and particularly respiratory infections (Gould & Drey, 2008).

A study that was conducted by Asadollahi, M et al in Tabriz, it was shown that the nurses had knowledge about healthcare at average level and 68% of them needed training in this field (Asadollahi et al., 2015). The survey of L Malekmakan et al indicated that most of nurses had quantitative knowledge about hand hygiene so and they did not assume it necessary to execute the care-giving to patient during this process (Malekmakan et al., 2008). MB Shinde et al examined the performance of knowledge and attitude of nurses in India and showed that most of them had medium knowledge, poor attitude, and good performance regarding hand hygiene (Shinde & Mohite, 2014).

Studies have showed that the rate of prevalence of Nosocomial infections in Asia is at high level (25). The studies conducted in Iran indicate that the knowledge, attitude, and performance of nurses have cleared contradictory results. Some of them indicated the suitable level of knowledge, attitude, and performance of nurses while some studies show the weakness of these factors in nurses. This study has been carries out in order to analyze knowledge, attitude, and performance of nurses regarding hand hygiene

## 2. Materials and Methods

This cross-sectional study was conducted on 200 (of 240) nurses in hospitals of Kerman city (Bahonar, Hazrat Fatemeh, and Shefa) in Iran. The nurses were the given studied population. The inclusion criteria were having at least three months working background in this sector. To collect data, the accessible sampling technique was utilized. The exclusion criteria were disagreement for participation in this study.

### 2.1 Instruments

The research-made questionnaire was used for data collection. This questionnaire was composed of two sections. The first section: demographic specifications including age, gender, educational status, work background and type of sector. The second section comprises of three sections. The first part measured knowledge of participants about hand hygiene based on giving answer to 10 items. In which they answered yes and no to them. The no-answer is given zero score and score 1 belongs to answer-yes. The method of evaluation of knowledge variable was to give score 0-4 (low), 4-7 at average level, and 7-10 scores to assess high level. The second part was specified to measurement of nurses attitude about hand hygiene and it was composed of 10 items in which the questions were answered by Likert 5-scale spectrum (strongly agree (5), agree (4), no comment (3), disagree (2), and strongly disagree (1)). The attitude was evaluated by considering score (10-23) as low, score (24-36) as average, and score (37-50) as high. Third section of included performance of nurses regarding hand hygiene was measured within the trend of nursing care-giving by nurses in which knowledge (awareness) and attitude consisted of 10 questions and they were answered according to Likert five-scale spectrum (always (5), mostly (4), a half of cases (3), some cases (2), and never (1)). The method of evaluation of the level of performance in nurses was the same as for attitude where it was considered by score (10-23) as low, scores (24-36) as average, and score (37-50) as high. The related essays in this field and close to goal of study were used for preparation of questionnaire (Erasmus et al., 2010; Ghezeljeh et al., 2015; van de Mortel et al., 2001).

To collect data after coordinating with hospitals, Questionnaires were distributed among nurses each nurse had ten minutes to complete the questionnaire after completing the questionnaire was collected.

After comprehensive analysis of texts related to the goal of study, the questionnaire was distributed among 10 experts in the field of controlling Nosocomial infections and the comments of experts about relevance of questions were implemented for nursing field in order to verify validity. In order to confirm reliability of questionnaire, it was distributed among 15 nurses and reliability of knowledge, attitude, and performance was approved with Cronbach alpha as 0.83, 0.87, and 0.91, respectively.

### 2.2 Data Analysis

To describe demographic attributes for variables of attitude, knowledge (awareness), and performance, descriptive tests of frequency and percentage of frequency, mean, and standard deviation were utilized. Chi-Square was employed for study on relationship in fields of knowledge, attitude and performance of nurses with demographic specifications. The confidence interval was 95% and significance level of P-value was considered smaller than 0.05 (significant).

### 2.3 Ethical Considerations

The written consent letter was taken from nurses for participation in the study and the nurses were allowed to exit from study trend whenever they liked. The participants were ensured that their private names and specifications will never be disclosed. The written consent letter was taken from all participants.

## 3. Findings

Among 200 questionnaire forms, all 200 forms were filled out and returned. The rate of responsiveness was 100 percents. The age range of nurses was 20-57 years and their mean age was 32.7 years (SD = 4.6). Most of participants were male 173 (86.5%), were had BScN degree 161 (80.5%) and married ones 155 (70.5%). Most of nurses 77 (38.5%) had working experience (5-10 years). Concerning the ward of activity, the majority of nurses 58 (29%) was working intensive units (ICU, CCU, and dialysis). (Table 1)

**Table 1:** Demographic specifications

Variables	Mean ± SD
Age	32.7 ± 4.6
	Frequency (frequency percentage)
Gender	
Female	27 (13.5%)
Male	173 (86.5%)
Marital status	
Single	45 (22.5%)
Married	155 (70.5%)
Education level	
Diploma	24 (12%)
BScN	161 (80.5%)
MScN	15 (7.5%)
Ward	
ICU, CCU	58 (29%)
Medical	13 (6.5%)
Surgical	30 (15%)
Pediatrics	26 (13%)
Infections	13 (6.5%)
Gynecology	14 (7%)
Working background (years)
0-5	68 (34%)
5-10	77 (38.5%)
11-20	34 (17%)
More than 20	21 (10.5%)

The mean and standard deviation scores of nurses’ knowledge about hand hygiene were 8.1 ± 1.04 among nurses. Most of nurses 149 (74.5%) had good knowledge about hand hygiene (Table 2). The nurses mainly selected the proper answers in the following items: hand hygiene should be done upon arrival and departure from isolation room; they should take off ring, wrist watch, and bracelet of their hands before start of scrubbing for surgery (196, 98%). But they had chosen wrong answers mainly in the following questions: execution of hand hygiene is not only necessary after doing official activities (137, 68.5%); using antiseptics is necessary before wearing gloves and after taking it off (55, 27.5%); the hot water should not be used for washing hands in healthcare centers since it may increase risk of dermal irritation (46, 23%). (Table 3)

**Table 2:** Frequency and percentage frequency and mean values of variables knowledge, attitude, and performance of nurses about hand hygiene

	Ranking	Quantity	Percent	Mean ± SD
Knowledge	Low	1	0.5	8.11 ± 1.04
	Average	50	25	
	High	149	74.5	
Attitude	Low	1	0.5	42.04 ± 2.11
	Average	55	27.5	
	High	141	70.5	
Performance	Low	2	1	44.12 ± 3.41
	Average	23	11.5	
	High	175	87.5	

**Table 3:** Frequency distribution and percentage of frequency of nurses’ answers about their knowledge regarding hand hygiene

Questions	Answers			
	Yes		No	
	N	%	N	%
The ring, wrist watch, and bracelet shall be taken off hands before starting scrubbing for surgery.	193	96.5	7	3.5
The hand hygiene is not necessary only after doing official tasks.	63	31.5	137	68.5
The hand hygiene shall be done before taking electrocardiogram from the patients.	173	68.5	27	13.5
The hand hygiene shall be executed upon arrival and departure from isolation room.	196	98	3	1.5
The hot water should not be used for washing hands in healthcare centers since it may increase risk of dermal stimulation.	153	76.5	46	23
Compared to other facilities, the alcoholic-based detergents for hand may more efficiently reduce the number of the existing bacteria in hand.	166	83	34	17
The hands should be rubbed together when rubbing them with alcoholic detergents for 60s.	157	78.5	43	21.5
Using antiseptics will be necessary before wearing gloves and after taking it off.	145	72.5	55	27.5
The ring, wrist watch, and bracelet shall be taken off hands before starting scrubbing for surgery.	196	98	4	2
The glove should be replaced during care-giving to the patient upon displacement from contaminated part to a clean part.	185	92.5	15	7.5

The mean score and standard deviation of nurses’ attitude about hand hygiene was 42.04 ± 2.11. Most of nurses 141 (70.5%) took good attitude toward hand hygiene (Table 2). Those items toward which the nurses mainly take positive attitude were as follows: I am tasked to act as a model for other healthcare personnel about hand hygiene; the existing infectious diseases in health care-giving environment may threaten my life and occupation (186, 96%); Execution of hand hygiene may reduce the related medical costs to Nosocomial infections under the recommended conditions (182, 91%). The item about which most of nurses had no comment was in that: I think one could follow the medical service officials in order to make decision for execution and or non-execution of hand hygiene (44, 22%). The items for which the nurses took negative attitude included: It is more important for me to fulfill perfectly my tasks than doing hand hygiene when the given ward is busy (90, 45%) and I could not always do hand hygiene under the recommended situations because of preference of my patients’ requirements (68, 34.5%). (Table 4)

**Table 4:** Frequency distribution and percentage of frequency of nurses’ attitude toward hand hygiene

Nurses’ attitude toward hand hygiene	Agree and strongly agree N (%)	No comment N (%)	Disagree and strongly disagree (N (%)
I am tasked to act as a model about hand hygiene for other healthcare personnel.	186 (93)	6 (12)	2 (1)
It is more important for me to fulfill perfectly my tasks than doing hand hygiene when the given ward is busy.	73 (36.5)	37 (18.5)	90 (45)
Execution of hand hygiene may reduce mortality of patients under the recommended conditions.	174 (87)	22 (11)	4 (2)
Execution of hand hygiene may reduce the related medical costs to Nosocomial infections under the recommended conditions.	182 (91)	8 (4)	8 (4)
I could not always do hand hygiene under the recommended situations because of preference of my patients’ requirements.	111 (55.5)	19 (9.5)	69 (34.5)
Prevention from the acquired infections is deemed as one of valuable roles for personnel of healthcare services.	179 (89.5)	14 (7)	6 (3)
I think one could follow the medical service officials in order to make decision for execution and or non- execution of hand hygiene.	144 (72)	34 (17)	21 (10.5)
The existing infectious diseases in health care-giving environment may threaten my life and occupation.	186 (93)	9 (4.5)	12 (6)
I think I have potential to change poor performances regarding hand hygiene in my workplace.	143 (71.5)	44 (22)	11 (5.5)
The hand hygiene is assumed as a habit in my personal life.	171 (85.5)	21 (10.5)	7 (3.5)

The mean scores and standard deviation in performance of nurses about hand hygiene was 44.12 ± 3.41. (Table 2) so that it signifies their good performance regarding hand hygiene. The results showed that most of nurses 175 (87.5%) had good performance about hand hygiene (Table 2). The item that was always observed by nurses included: washing hand after going toilet (187, 93.5%); the item that was mainly observed by them was washing hand before entry in isolation room (51, 25%); the item that was observed by them in a half of cases was washing their hands before invasive measures (27, 13.5%); the item that was observed by them in some cases was washing their hands after touching with patient’s skin (21, 10.5%); and the item that was never observed by nurses was related to it before care-giving of wound (15, 7.5%). (Table 5)

**Table 5:** Frequency distribution of nurses’ performance upon the needed times for doing hand hygiene

Performance	Answers				
	Never N (%)	Some cases N (%)	A half of cases N (%)	Mainly N (%)	Always N (%)
After going toilet	2 (1)	1 (0.5)	4 (2)	6 (3)	187 (93.5)
Before care-giving for wound	15 (7.5)	10 (5)	15 (7.5)	45 (22.5)	115 (57.5)
After care-giving for wound	2 (1)	2 (1)	9 (4.5)	34 (17)	153 (76.5)
After touching potentially dirty objects	0 (0)	2 (1)	3 (1.5)	28 (14)	167 (83.5)
After touching blood or body fluids	1 (0.5)	2 (1)	3 (1.50	23 (11.5)	171 (85.5)
After making invasive measures	0 (0)	1 (0.5)	7 (3.5)	38 (19)	154 (77)
Before entry in isolation room	4 (2)	8 (4)	24 (12)	51 (25.5)	113 (65.5)
After touching patient’s skin	1 (0.5)	21 (10.5)	27 (13.5)	37 (18.5)	114 (57)
After existing from isolation room	5 (2.5)	10 (5)	22 (11)	45 (22.5)	118 (59)
Before making invasive measures	2 (1)	18 (9)	27 (13.5)	46 (23)	107 (53.5)

The results of Chi-Square did not significantly difference among gender, education from knowledge and attitude of nurses (p>0.5) but a statistical significant relationship was seen among working background with attitude and performance of nurses (p<0.05). Chi-squire test did not significantly correlation between knowledge, attitude and performance of nurses about hand hygiene (P>0.05).

## 4. Discussion

The present study showed that most of nurses (195, 74.5%) had good knowledge about hand hygiene. Due to type and period of their relation with patients such knowledge is necessary. The study of Tabrizi et al. showed that the nurses had good knowledge about hand hygiene. Similarly, this study indicated that the level of nurses’ knowledge is appropriated in the field of Nosocomial precautions particularly about methods of transferring infection and proper time for doing hand hygiene (Asadollahi et al., 2015). In Pittet D et al study it was shown that most of them had good knowledge and this verifies the results of our studies (Pittet, 2001).

But the results of study done by Mahadeo B et al. on nurses and nursing students in India showed that the nurses possessed knowledge about hand hygiene at medium level so that the reason for this difference may be due to different studied population (Shinde & Mohite, 2014). The study of Akyol et al. indicated that nursing students had quantitative knowledge about hand hygiene that is unlike our study. This may be due to the learning environment of samples (Akyol, 2007). Likewise, the other study showed the majority of nurses have acquired score less than six scores out of 12 scores and this indicated their little knowledge about hand hygiene (Ghezeljeh et al., 2015). In our studies, most of nurses had better knowledge in the fields of washing hands before and after exiting from isolation room and method of washing hands but regarding this point that washing hand is not only necessary out of non-official times and wearing gloves is only necessary upon time of touching patient (Tanwir, 2012; Tavolacci et al., 2008), they had lesser knowledge. Unlike study of Najafi et al. that was carried out on nurses, the nurses mainly selected proper items of ‘hand hygiene after doing official activities and before taking electrocardiogram’ but they had lesser knowledge about questions of proper hand washing and scrubbing. This difference may be due to the study which was conducted on students in Saudi Arabia that showed the score of their knowledge was 38 out of 53 and this was higher than average level (Ghalya & Ibrahim, 2014).

The results of our study indicated that most of nurses (141, 70.5%) took positive attitude toward hand hygiene. The review on different studies shows that the nurses have positive approach to hand hygiene (O’Boyle, Henly, & Duckett, 2001; Pessoa-Silva et al., 2005; White et al., 2015). Similar to study of Najafi et al that showed many nurses took positive attitude toward hand hygiene and reducing of Nosocomial infections was one of the roles for healthcare personnel so by the aid of hand hygiene the hospital costs can be efficiently reduced. In a study, similar to our survey, which was carried out in Tehran, conversion of them into a model about hand hygiene among their colleagues was the item that received the maximum number of proper answer (Ghezeljeh et al., 2015). In a study that was done in Saudi Arabia, 78.82% of students had positive approach to hand hygiene (Ghalya & Ibrahim, 2014). This may be due to little knowledge and lack of experience in students regarding these techniques.

But the study of Shinde and Mohite (2014) showed that the nurses and physicians took negative attitude toward hand hygiene while the nurses had better attitude than physicians. Similarly, the nurses argue that it always necessitates learning about hand hygiene and such a positive attitude shows the nurses are necessarily ready to acquire the required knowledge and practical skills about hand hygiene.

The results of this study indicated that most of nurses had good performance about hand hygiene that may represent holding appropriate training courses in these hospitals. But in a study that was conducted in Saudi Arabia, it was shown that the majority of research population was poor performance in hand hygiene (Ghalya & Ibrahim, 2014). The reason for such poor performance may be due to high working stress, negative attitude, little knowledge, change in work-shifts, infection risk in patients, and allergy to antiseptics (Erasmus et al., 2010). One of the limitations in our study is different impressions of respondents of questions in this questionnaire that may cause diversion in these results. The other limitation was sample size where only 200 participants had completed questionnaire forms out of total 250 cases.

## 5. Conclusion

According to the given results, the nurses are placed at appropriate level in terms of knowledge, attitude, and performance but there are some fields in which the nurses had lesser knowledge in these areas as a result it seems more necessarily to increase their knowledge by holding training classes and courses, especially in cases such as appropriate time for observance of healthcare and for hand hygiene in which nurses had less knowledge. The nurses take positive attitude toward hand hygiene and this signifies their readiness for learning better the clinical principles and guidelines about hand hygiene that was prepared by WHO, American Center for Control and Prevention of Diseases, and regional healthcare organizations. But in order to improve nurses’ performance more than ever, it is recommended to prepare and execute an applied plan including appropriate principles, procedures, theoretical and practical manual. Whereas this study has been carried out within a limited sample size thus it is suggested to do it with greater sample size. Likewise, it is suggested to address the impact of cultural dimensions of nursing community and their effects on better performance regarding hand hygiene in the future studies.
